# Assessment of a bedside test for N-terminal pro B-type natriuretic peptide (NT-proBNP) to differentiate cardiac from non-cardiac causes of pleural effusion in cats

**DOI:** 10.1186/s12917-017-1319-6

**Published:** 2017-12-20

**Authors:** Gabriel Wurtinger, Estelle Henrich, Nicolai Hildebrandt, Nicola Wiedemann, Matthias Schneider, Esther Hassdenteufel

**Affiliations:** Small Animal Clinic (Internal Medicine), Justus-Liebig-University Giessen, Frankfurter Strasse 126, 35392 Giessen, Köln, Germany

**Keywords:** Feline, Pleural effusion, Biomarker, Point-of-care test

## Abstract

**Background:**

Cats with pleural effusion represent common emergencies in small animal practice. The aim of this prospective study was to investigate the diagnostic ability of a point-of-care ELISA (POC-ELISA) for the measurement of N-terminal pro B-type natriuretic peptide (NT-proBNP) to differentiate cardiac from non-cardiac disease in cats with pleural effusion. The sample material for use of this rapid test was either plasma or diluted pleural effusion.

Twenty cats with moderate to severe pleural effusion were prospectively recruited. The cats were grouped into two groups, with or without congestive heart failure (CHF; N-CHF), after complete work-up. Blood and effusion were collected in EDTA tubes. Plasma and pleural effusion supernatants were transferred into stabilizer tubes and frozen. POC-ELISA for NT-proBNP was performed with plasma and diluted effusion (1:1). Quantitative NT-proBNP measurement was performed in plasma and diluted and undiluted effusions.

**Results:**

Six cats were assigned to the CHF group. Of the 14 cats in the N-CHF group, 6 had concurrent cardiac abnormalities that were not responsible for the effusion. For the detection of CHF, the test displayed respective sensitivities and specificities of 100% and 79% in plasma and 100% and 86% in diluted pleural fluid. Receiver operating characteristic (ROC) analysis for quantitative NT-proBNP measurement of plasma and diluted and undiluted pleural effusions displayed areas under the curve of 0.98, sensitivities of 100% and specificities of 86%. The optimum cut-off was calculated at 399 pmol/l in plasma and 229 pmol/l in the diluted effusion and 467 pmol/l in the undiluted effusion.

**Conclusions:**

POC-ELISA for NT-proBNP in both plasma and diluted pleural effusion was suitable to differentiate cardiac from non-cardiac causes of feline pleural effusion. According to our results, use of pleural effusion is feasible, but dilution of the effusion before measurement seems to improve specificity.

**Electronic supplementary material:**

The online version of this article (10.1186/s12917-017-1319-6) contains supplementary material, which is available to authorized users.

## Background

Due to severe dyspnea, cats with pleural effusion are commonly presented to an emergency service. In addition to feline infectious peritonitis (FIP), pyothorax, neoplasia and idiopathic chylothorax, congestive heart failure (CHF) represents a common underlying disease [1, 2]. Thoracic radiographs are commonly performed but do not enable differentiation between cardiac and non-cardiac causes in cases where moderate to severe pleural effusion is present [3]. Echocardiography is helpful, but the availability of this diagnostic tool is limited due to the need for trained staff and specialized equipment. This makes decisions about the administration of further cause-specific therapies (especially diuresis) difficult in these patients.

Cardiac biomarkers have been used with increasing frequency to differentiate cardiac from non-cardiac dyspnea in small animal medicine. Studies have investigated the utility of NT-proBNP as a biomarker in cats [4–11]. The synthesis of its precursor, proBNP, increases in response to myocardial wall stress. ProBNP is cleaved by proteases into the biologically active form BNP and the inactive NT-proBNP [12]. NT-proBNP is preferred as a biomarker due to its longer plasma half-life and higher plasma concentrations, which was proven experimentally in sheep [13] and is presumed to be the same in other animals.

First generation enzyme-linked-immunosorbent assay (ELISA) was used to measure the concentration of NT-proBNP in both plasma [14, 15] and pleural fluid [15] to differentiate between cardiac and non-cardiac causes of pleural effusion in cats. This assay is not available as an in-house test, and the sample has to be sent to an external laboratory. A major disadvantage of this approach is the delay of up to 72 h in obtaining the test results, which makes this approach unsuitable for emergency situations. Recently, a second-generation ELISA was developed and is available as a quantitative ELISA and as a semiquantitative point-of-care (POC) test. The use of this test was first reported in a publication investigating the detection of moderate to severe occult cardiomyopathy in cats [16] and it was recently described for diagnosing CHF in feline population with pleural effusion [17]. Specificity to differentiate CHF versus non-CHF in cats with pleural effusion was low with 64.7% for the POC test, which is likely due to a low transition point from negative to positive of 150 to 200 pmol/l according to the study [17]. Dilution of the pleural effusion to reduce the number of false positive results could be a method to improve specificity. The aim of our study was to investigate the usefulness of diluted pleural samples for measurement of NT-proBNP POC-ELISA to differentiate cardiac from non-cardiac causes of moderate to severe pleural effusion in cats. The secondary aim was to confirm the results of the previous study about the use of quantitative and POC-Test in plasma in a more severely diseased group of cats.

## Methods

Cats with pleural effusion were consecutively included in the prospective study between March 2013 and April 2014. The following inclusion criteria had to be fulfilled:

1. Presence of relevant clinical signs leading to admission to the intensive care unit (ICU);
2. Radiographic confirmation of moderate to severe pleural effusion (obliterated cardiac and diaphragmatic borders) [18];
3. Echocardiography request by the primary clinician.

Radiography was performed either by the referring veterinarian or at presentation to the clinic. Initial echocardiography was performed either by a board-certified cardiologist or under his supervision by a cardiology resident or by a board-certified specialist in critical care to confirm the presence of pleural effusion and to classify the patient as being in congestive heart failure (CHF) or having a non-cardiogenic cause of pleural effusion (N-CHF). The group assignment (CHF versus N-CHF) was performed by a board-certified cardiologist according to full echocardiogram, history, physical examination and any additional work-up. Patients with CHF underwent complete echocardiographic examination (including Doppler-echocardiography and pulsed wave Tissue Doppler Imaging), ECG and Doppler-based blood pressure measurement. Underlying heart disease was classified according to the echocardiography results by a board-certified cardiologist. Cardiomyopathies were divided in 5 possible types according to published criteria [19]: hypertrophic cardiomyopathy (HCM), restrictive cardiomyopathy (RCM), dilated cardiomyopathy (DCM), arrhythmogenic right ventricular cardiomyopathy (ARVC) and unclassified cardiomyopathy (UCM). Main echocardiographic criteria were: M-mode derived diameter of left ventricular free wall and septum (cut off >6.0 mm) for diagnosis of HCM [20], right ventricular diameter (cut off >8.3 mm) [20] for right ventricular dilation; maximum left atrial dimension in B-mode in a long axis view (cut off >15.8 mm) [21] for left atrial dilation. Right atrial size was classified subjectively.

Concurrent cardiac abnormalities in patients with non-cardiac causes of pleural effusion (N-CHF group) were also recorded. Thoracocentesis was performed in all cats followed by pleural fluid analysis and cytological examination. In addition, a complete blood count and a biochemical panel were performed in all cats. After initial stabilization, the diagnostic work-up was performed based on the assignment to one of the two groups. In the N-CHF group, diagnostic investigation into the underlying non-cardiogenic disease was tailored to the individual patient. This included laboratory investigations (pleural fluid analysis, cytology of fine needle aspirates, blood examinations) and diagnostic imaging modalities (radiography, ultrasound).

Blood for NT-proBNP measurement was collected by venipuncture in EDTA-tubes. Pleural fluid samples were also placed in EDTA tubes and subsequently treated in the same way as blood samples. Sampling of blood and pleural effusion had a maximum time difference of 1 h. All samples in the EDTA tubes were centrifuged at 9000 x g for 1 min within 30 min of collection. EDTA plasma and EDTA pleural fluid supernatant were then placed in specific tubes containing a protease inhibitor mixture^1^ and were frozen at −20 °C for up to one week and at −80 °C thereafter.

A POC-ELISA^2^ for feline NT-proBNP was performed on each sample; this test had a transition point from negative to positive of 200 pmol/l according to the manufacturer. Undiluted plasma and pleural fluid samples diluted with an equal amount of NaCl 0.9% were analyzed. The POC-ELISA was performed by one investigator according to the manufacturer’s guidelines.^3^ Three drops of the sample and five drops of the assay conjugate were mixed in a tube, and the mixture was then poured into the POC-ELISA sample well. When the sample-conjugate mixture reached the indicator window, the device was activated by the operator. After 10 min of incubation, the color density of the sample and the reference spot were evaluated. The analysis was performed using an automated optical density reader^4^ followed by visual inspection. The reader gives a result classifying the test as either normal or abnormal according to relative optical density. Immediately afterwards digital images of the POC-ELISA were obtained using a standard scanner at 600 dots per inch. These pictures in the original size of the POC ELISA were inspected by one investigator blinded to the results of echocardiography and interpreted according to the manufacturer’s guidelines for visual inspection. The results were considered normal if the sample spot was lighter than the reference spot and abnormal if it was equal or darker.

In addition, the following samples were shipped frozen to a commercial laboratory^5^ and were quantitatively analyzed using a previously described feline NT-proBNP test^6^ [22] by laboratory technicians blinded to the final diagnosis: EDTA plasma, pleural fluid supernatant, and pleural fluid supernatant diluted 1:1 with 0.9% saline solution refrozen after POC analysis. Samples with NT-proBNP concentrations below the lower detection limit (24 pmol/l) were reported as 17 pmol/l (lower detection limit/$\sqrt{2}$) [23] for statistical purposes. Samples with NT-proBNP concentrations greater than the upper detection limit of the test (1500 pmol/l) were diluted 1:2 with a feline NT-proBNP diluent to obtain a result.

To evaluate the effect of dilution of the effusion samples, the ratio between the NT-proBNP concentration in the paired undiluted and diluted samples was calculated for all samples with NT-proBNP concentrations within the detection limit of the assay.

Due to the small group size of the CHF group, non-Gaussian distribution was assumed. Descriptive statistics included frequencies for categorical variables and median and range for continuous variables. The data were compared between groups (CHF versus N-CHF) using the Mann-Whitney U test. Proportions were compared using Fisher’s exact test. The sensitivity and specificity and their 95% confidence interval (CI) for diagnosing CHF were calculated. Quantitative NT-proBNP values were graphically depicted as scatter plots of individual data points and were analyzed with receiver operator characteristic (ROC) analysis to determine the ability of NT-proBNP to diagnose CHF. The area under the curve (AUC) was used as a summary measure and quantification of diagnostic accuracy for NT-proBNP to predict CHF. The cut-off value was chosen based on the highest Youden index (Y = sensitivity + specificity - 1) [24]. Statistical analysis was performed using commercially available software.^7^ *P* values ≤ 0.05 were considered significant.

## Results

Twenty cats were included in this study. Fourteen cats were classified as N-CHF and 6 as CHF. In the N-CHF patients, the following underlying diseases were diagnosed: neoplasia (*n* = 7), septic pyothorax (*n* = 3), hemothorax caused by chronic pleuritis (*n* = 1), feline infectious peritonitis (*n* = 1), pancreatitis (*n* = 1) and steatitis (*n* = 1). Six N-CHF patients had the following concurrent cardiac abnormalities: right atrial and ventricular dilation (*n* = 4), HCM (*n* = 1), and UCM (*n* = 1). A board-certified cardiologist judged that these changes were not severe enough to cause pleural effusion. In cats with CHF, the diagnoses were: HCM (*n* = 2), UCM (n = 2), DCM (*n* = 1), double chambered right ventricle (n = 1).

Patient data are shown in Table 1, and echocardiographic data in Table 2. There were no significant differences concerning sex distribution, age or body weight between the groups. Additionally, no significant differences were found regarding heart and respiratory rates. Body temperature was significantly lower in cats with CHF (*p* = 0.043).

**Table 1:** Summary of the data in two groups of cats without (N-CHF) or with (CHF) congestive heart failure

	N-CHF (*n* = 14^a^)	CHF (*n* = 6)	*p*-value
Breeds	DSH (8) Crossbreed (2) Maine Coon (1) Siamese (1) Persian (1) Balinese (1)	DSH (4) Maine Coon (1) Bengal (1)	–
Sex (m/f)	6/8	3/3	1.0
Age (year)	8.0 (0.8–12.9)	8.4 (2.3–16.9)	0.77
Body weight (kg)	4.2 (2.6–6.0)	4.8 (2.9–7.7)	0.41
Heart rate (/min)	188 (160–240)^a^	195 (150–220)	0.93
Respiratory rate (/min)	60 (36–100)^a^	60 (28–80)	0.64
Temperature (°C)	38.7 (36.5–40.0)^a^	37.2 (36.3–38.2)	0.043
Pathological cardiac auscultation	3/13^a^	3/6	0.32
NT-proBNP (pmol/l) (median, range) Pleural effusion 1:1 diluted	47 (17–329)	924.5 (249–1162)	0.0011
NT-proBNP (pmol/l) (median, range) Pleural effusion	108.5 (17–732)	1875 (509–2077)	0.0011
NTproBNP (pmol/l) (median, range) Plasma	144.5 (17–552)	1698 (459–1942)	0.0011

^a^clinical data (heart rate, respiratory rate, temperature, and auscultation) were missing in one cat due to computer problems

*DSH* Domestic Short Hair, *m* male, *f* female

**Table 2:** Echocardiographic findings in two groups of cats without (N-CHF) or with (CHF) congestive heart failure

		N-CHF (*n* = 14)	CHF (*n* = 6)
2D long axis	LAD (mm)^a^	14.0 (8.9–22.0)	19.0 (8.2–41.0)
	RA increased^b^	4	3
M-mode long axis	IVSd (mm)^a^	4.6 (3.7–6.7)	4.9 (2.8–7.9)
	LVWd (mm)^a^	5.0 (3.4–7.7)	5.2 (3.1–8.6)
	RVDd (mm)	5.9 (2.1–11.0)	7.5 (3.7–13.0)

*LAD* left atrium systolic diameter, *RA* subjective right atrium size, *IVSd* interventricular septum diastolic diameter, *LVWd* left ventricular wall diastolic diameter; ^a^ = median (range); ^b^ number of patients

In both groups, there was one patient each with heart murmur, gallop rhythm and arrhythmia. The proportion of cats with abnormal auscultation findings was not significantly (*p* = 0.32) different between the N-CHF (3/13; 23%) and CHF groups (3/6; 50%).

The sample storage times ranged between 2 and 409 days. For both plasma and diluted pleural effusion, there was no difference in the interpretation between visual inspection and automatic reading in the POC analysis.

**Fig. 1:**
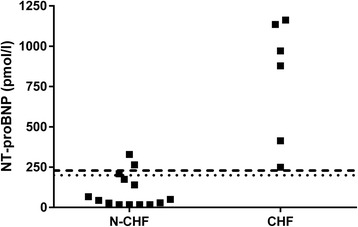
Quantitative NT-proBNP values in diluted pleural effusion. There was a significant difference (*p* = 0.0011) between the N-CHF and CHF cats. N-CHF = cats without congestive heart failure; CHF = cats with congestive heart failure. The dashed line represents the calculated best cut-off of 229 pmol/l. The dotted line represents the transition point of the POC-ELISA of 200 pmol/l

When testing the diluted pleural effusion, 2/14 (14%) cats in the N-CHF group and 6/6 (100%) cats with CHF were positive on the POC test, which led to a sensitivity of 100% (95% CI: 54.1% to 100%) and a specificity of 85.7% (95% CI: 57.2% to 98.2%) for a diagnosis of CHF. Likewise, the median NT-proBNP concentration in diluted pleural effusion (Fig. 1) was significantly (*p* = 0.0011) different between the N-CHF and CHF cats (47 pmol/l, range: 17–329 pmol/l versus 924.5 pmol/l, range 249–1162 pmol/l). The AUC was 0.98 (95% CI: 0.92–1.00), and the optimal cut-off of 229 pmol/l had a sensitivity of 100% (95% CI: 54.1% to 100%) and a specificity of 85.7% (95% CI: 57.2% to 98.2%). The use of 200 pmol/l (transition point of the POC test) as a cut-off for quantitative testing led to a sensitivity of 100% (95% CI: 54.1% to 100%) and a specificity of 78.6% (95% CI: 49.2% to 95.3%).

**Fig. 2:**
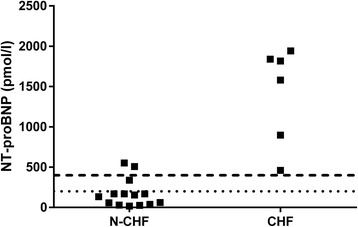
Quantitative NT-proBNP values in plasma. There was a significant (*p* = 0.0011) difference between N-CHF and CHF cats. N-CHF = cats without congestive heart failure; CHF = cats with congestive heart failure. The dashed line represents the calculated best cut-off of 399 pmol/l. The dotted line represents the transition point of the POC-ELISA of 200 pmol/l

Three out of 14 (21%) cats in the N-CHF group and 6/6 (100%) cats with CHF were positive on the POC test when plasma samples were evaluated, which resulted in a sensitivity of 100% (95% CI: 54.1% to 100.0%) and a specificity of 78.6% (95% CI: 49.2% to 95.3%) for the diagnosis of CHF for the cause of pleural effusion. The quantitative values of plasma NT-proBNP (Table 1, Fig. 2) were significantly (*p* = 0.0011) different between the N-CHF (median: 144.5 pmol/l; range: 17–552) and CHF cats (median 1698 pmol/l; 459–1942 pmol/l). The AUC was 0.98 (95% CI: 0.92–1.00), and the optimal cut-off of 399 pmol/l gave a sensitivity of 100% (95% CI: 54.1% to 100%) and a specificity of 85.7% (95% CI: 57.2% to 98.2%).

Three cats in the N-CHF group with concurrent cardiac abnormalities (1 with HCM, 2 with right heart dilation) had quantitative test results higher than 200 pmol/l in both diluted pleural effusion and in the plasma (s. Figs. 1 and 2). All three cats had positive POC results in plasma, and two of them also had positive results in diluted pleural effusion. The diluted pleural effusion sample that tested negative by POC-ELISA had a NT-proBNP concentration of 209 pmol/l, which is just above the supposed transition point of the POC-test.

The quantitative NT-proBNP measurements were also performed with undiluted pleural effusion. Cats in the N-CHF group had significantly (*p* = 0.0011) lower median NT-proBNP values than did cats in the CHF group (108.5 pmol/l, range: 17–732 versus 1875 pmol/l, range 509–2077 pmol/l). The AUC was 0.98 (95% CI: 0.92–1.0), and the cut-off of 467 pmol/l yielded a sensitivity of 100% (95% CI: 54.1% to 100%) and a specificity of 85.7% (95% CI: 57.2% to 98.2%). The use of 200 pmol/l as a cut-off value gave a sensitivity of 100% and a specificity of 64.3%.

In 12 cats, the NT-proBNP concentrations in diluted and undiluted effusion samples were between the lower and upper detection limits of the quantitative test. In these patients, the ratios between the paired samples of the quantitative NT-proBNP measurements in undiluted and diluted pleural effusion were calculated and had a mean of 2.23 (standard deviation 0.23).

## Discussion

In the present study, POC-ELISA for NT-proBNP was able to differentiate between cardiac and non-cardiac causes of pleural effusion in cats using either plasma or diluted pleural effusion samples. In addition, there was significant agreement between automated and optical evaluation as described earlier [16], which makes this test helpful for routine use in general practice.

Our study supported recent results reported with plasma samples [17] as the POC-ELISA performed comparably in both studies with excellent sensitivity (100% (own study) versus 95.2% [17]) and good specificity (78.6% (own study) versus 87.5% [17]). Dilution of the pleural effusion samples resulted in a high sensitivity as described for undiluted samples [17] (100% each). The previous study that did the measurement in undiluted pleural effusion had a low specificity of 64.7% (95% CI: 41.3% to 82.7%) [17]. Our results showed a specificity 85.7% (95% CI: 57.2% to 98.2%) in diluted samples. Because there was a wide overlap between the confidence intervals, the significance of this difference has to be proven in a larger number of cases.

Our study design was similar to earlier studies using the first generation test [14, 15] and to a previous study using the second generation ELISA [17]. The main difference in our study is the use of diluted pleural effusion. The rationale for dilution was the need to reduce NT-proBNP concentrations in the pleural fluid. Two studies [15, 17] showed higher concentrations of NT-proBNP in pleural effusion compared with plasma, and approximately one-quarter [15] or more [17] of the pleural fluid samples in the non-cardiac cases had NT-proBNP values above the reported transition point of the POC test (200 pmol/l). The dilution resulted in slightly lower concentrations of NT-proBNP in the diluted material than expected by calculation (ratio between paired samples of undiluted and diluted effusion of 2.2 instead of 2.0). This might have been caused by sample handling and degradation or a matrix effect of the diluent. Finally, the dilution seemed to be effective, as 5/14 non-cardiac cats had values above 200 pmol/l in undiluted samples in contrast to 3/14 cats in diluted samples. None of the cardiac cats had values lower than 200 pmol/l after dilution; this trend has to be proven in a larger group of cats. The specificity for quantitative measurement of diluted pleural effusion at a cut off at 200 pmol/l was 78.6%, and this was slightly higher for the POC test (85.7%). The reason for this was that one cat that had a NT-proBNP concentration of 209 pmol/l tested negative with the POC test. It seems that the transition point in our batch lay between 209 pmol/l (highest quantitative value with negative POC test) and 249 pmol/l (lowest quantitative value with positive POC test). Compared with an earlier study with an approximate transition point of 150 pmol/l [16], this value was markedly increased. For future use, it seems desirable to know the switch point of the actually utilized batch to adapt the dilution factor.

The second generation quantitative NT-proBNP assay had good performance in the present as well as in a previous study [17]. The cut off values were higher in our study both in plasma (399 pmol/l versus 199 pmol/l) and in pleural effusion (467 pmol/l versus 240 pmol/l). This discrepancy may be explained by small group sizes and differences in selection of cases. While the other study [17] recruited all cases independent of the grade of pleural effusion, we only recruited symptomatic cases with moderate to severe effusion. With the corresponding cut offs in both studies, the sensitivity (95–100%) was excellent, and the specificity (77–86%) was good in both samples.

POC-ELISA and quantitative testing were both suitable as screening tests due to the high sensitivity in our study and the earlier study [17]. The limited specificity was probably caused by the number of cases with non-cardiogenic pleural effusion and concurrent cardiac abnormalities (6/14 in our study, more than 25% in the earlier study [17]). Many of the quantitative samples in our study (3/6 plasma and 4/6 pleural effusion samples) displayed concentrations above 200 pmol/l. Comparable results were not reported in the other study [17], but the findings may have been similar. We found NT-proBNP elevations in cats with severe as well as mild cardiac changes. In the more severely affected cases, the cause for NT-proBNP elevation was probably the cardiac wall stress itself, as described for cardiomyopathy in cats [4, 7, 11, 16], primary heart disease in dogs (e.g., DCM and valve disease) [25, 26] and pulmonary hypertension or embolism in dogs [27, 28]. In mildly affected cases, the elevation could be caused by concurrent cardiac or other disease processes as described for inflammatory conditions in humans [29], dogs^8^ [27] and cats [14] or malignancy in humans [30]. Increased NT-proBNP concentration has also been described in cats with advanced kidney disease [31], but none of our cats displayed comparable severe renal impairment.

### Study limitations

The major limitation of the study was the small number of patients, especially in the CHF group. Blood pressure was not routinely measured in non-cardiac cats.

Examination of the pleural fluid was not considered in the data interpretation but could aid in establishing the diagnosis, e.g., in cats with pyothorax.

POC-ELISA in pleural effusion was only evaluated in diluted but not undiluted samples. Comparison of undiluted and diluted pleural effusion with the POC-ELISA in the same patient could have shown more clearly the effect of dilution on the specificity.

We measured all of our samples on the POC-ELISA in a batch, and it would be better to test each patient immediately after sampling. This was not possible, as the test was not available at the beginning of patient recruitment.

To avoid any influence of any freeze-thaw cycles, simultaneous measurements of quantitative NT-proBNP would have been ideal.

## Conclusions

In this small number of patients using plasma and diluted pleural effusion, NT-proBNP POC-ELISA was appropriate to differentiate between cardiac and non-cardiac underlying disease in cats with moderate to severe pleural effusion. Using pleural effusion, 1:1 dilution with 0.9% NaCl is feasible and has the potential to improve the diagnostic accuracy of this test with this sample material.

## Additional file

Additional file 1: The table contains all information of the cats described and analyzed in this study (Signalement, Physical examination findings, Echocardiographic measurements, Diagnosis, Classification and NT-pro BNP values). (XLSX 13 kb)

## Abbreviations

ARVC: arrhythmogenic right ventricular cardiomyopathy
AUC: area under the curve
CHF: congestive heart failure
DCM: dilated cardiomyopathy
EDTA: Ethylendiamintetraacetate
ELISA: Enzyme-linked-immunosorbent assay
FIP: feline infectious peritonitis
HCM: hypertrophic cardiomyopathy
ICU: intensive care unit
NT-proBNP: N-terminal pro B-type natriuretic peptide
POC: Point-of-care
RCM: restrictive cardiomyopathy
ROC: Receiver operating characteristic
UCM: unclassified cardiomyopathy

## Footnotes

1 Cardiopet™ proBNP Transport Tube, IDEXX Laboratories, Westbrook, USA

2 SNAP Feline proBNP, IDEXX Laboratories, Westbrook, USA

3 SNAP Feline proBNP Test Product Insert, IDEXX Laboratories, Westbrook, USA

4 SNAPshot Dx®, IDEXX Laboratories, Westbrook, USA

5 Vet Med Labor GmbH, Division of IDEXX Laboratories, Ludwigsburg, Germany

6 Cardiopet™ proBNP, IDEXX Laboratories, Westbrook, USA

7 GraphPad Prism 5, GraphPad Software, Inc., San Diego, USA

8 K. Gommeren, I. Desmas, A. Garcia, L. Massart, C. Clercx, K. McEntee, D. Peeters, Cardiac troponin and natriuretic peptide in canine emergencies with a systemic inflammatory response syndrome, Proceedings 21st ECVIM-CA congress 2011: 249
